# Supplementary material for: Environmental enrichment preserves a young DNA methylation landscape in the aged mouse hippocampus
Source: Nat Commun. 2021 Jun 23;12:3892. doi: 10.1038/s41467-021-23993-1 (PMC8222384; doi:10.1038/s41467-021-23993-1)
Supplement: Supplementary file 1 — Supplementary information [file 41467_2021_23993_MOESM1_ESM.pdf]

Supplementary Information

Environmental enrichment preserves a young DNA methylation landscape in the aged mouse hippocampus

Sara Zocher[1,2], Rupert W. Overall[1,2], Mathias Lesche[2,3], Andreas Dahl[2,3], Gerd Kempermann[1,2*]

[1] German Center for Neurodegenerative Diseases (DZNE) Dresden, Tatzberg 41, 01307 Dresden, Germany

[2] Center for Regenerative Therapies Dresden (CRTD), Technische Universität Dresden, Fetscherstraße 105, 01307 Dresden, Germany

[3] DRESDEN-concept Genome Center c/o Center for Molecular and Cellular Bioengineering (CMCB), Technische Universität Dresden, Fetscherstraße 105, 01307 Dresden, Germany

*Corresponding author. E-mail: Gerd.Kempermann@dzne.de

Content:

Supplementary Figures 1-11 with figure legends

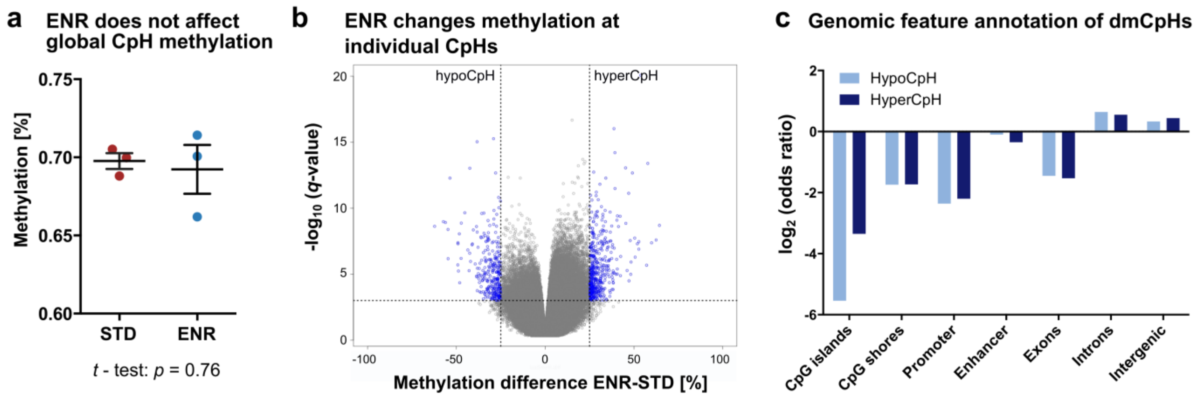

Supplementary Fig. 1

Environmental enrichment elicits locus-specific CpH methylation changes in the adult, non-aged dentate gyrus. DNA methylation profiles from 4.5 months old mice housed in ENR or STD for three months. **a**, ENR does not change global CpH methylation. Depicted are individual data points with mean ± SEM. **b**, Volcano plot depicting differentially methylated CpHs (dmCpHs) in blue. In total, 750 dmCpHs were detected (0.019 % of covered CpHs). Of those, 38.93 % were hypomethylated (hypoCpH) and 61.07 % were hypermethylated (hyperCpH) in ENR mice. **c**, Genomic distribution of hypoCpH and hyperCpH (adjusted $p < 0.001$ for CpG islands, CpG island shores, promoters, exons, introns; adjusted $p < 0.05$ for intergenic regions; enhancer: adjusted $p = 0.65$ for hypoCpH, adjusted $p = 0.26$ for hyperCpH).

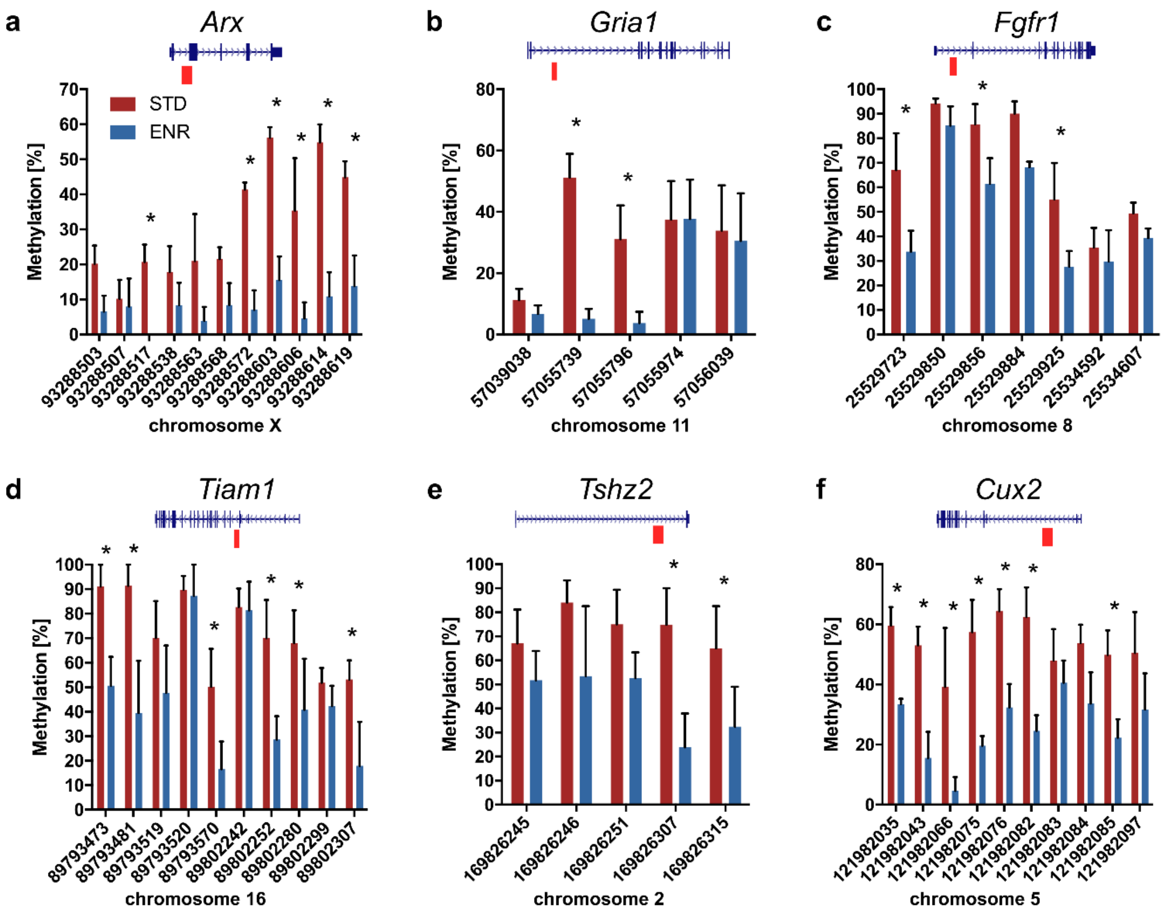

Supplementary Fig. 2

Representative ENR-induced DNA methylation changes at selected gene loci. **a-f,** Gene loci containing differentially methylated cytosines between dentate gyrus of young adult STD and three months-ENR housed mice as determined by RRBS (n = 3 individual mice per group). Depicted are mean methylation levels at individual CpGs with SEM. The relative position of the plotted genomic areas within the respective genes is indicated with a red bar above the graphs. * $q < 0.001$ (q-values from logistic regression with SLIM correction performed with Methylkit).

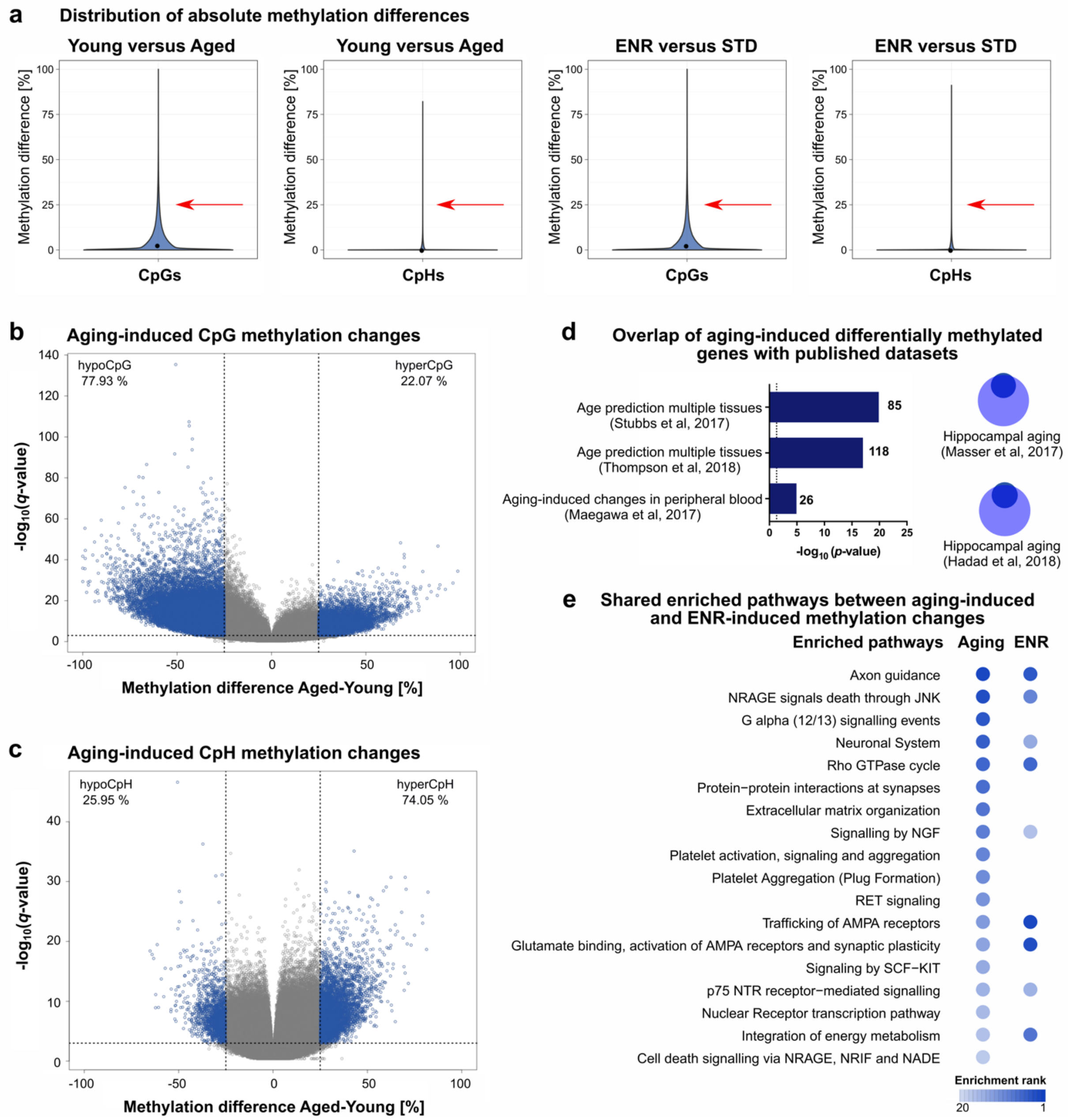

Supplementary Fig. 3

Age-related CpG and CpH methylation changes in the dentate gyrus. a, The distribution of the absolute DNA methylation differences at CpGs and CpHs justifies a threshold of 25 % (red arrow) for differentially methylated cytosines. Depicted are methylation differences between young and aged mice or aged ENR and aged STD mice as indicated. **b,** Volcano plot depicting CpGs where aging changed methylation in STD mice in blue (n = 5 for young mice; n = 7 for aged mice). In total, 5.51 % of all covered CpGs were dmCpGs. Age-related CpG hypomethylation (77.93 % of dmCpGs) is more prominent than CpG hypermethylation (22.07 % of dmCpGs). **c**, Aging changed methylation at 0.17 % of CpHs in the genome. The vast majority of dmCpHs were hypermethylated in the aged dentate gyrus (74.05 %). **d**, Enrichment of age-related differentially methylated genes (CpG and CpH context) from published datasets among genes with age-related CpG or CpH methylation changes in the dentate gyrus (*p*-values from hypergeometric tests; left). Venn diagrams (right) show overlap of age-related methylation changes found in this study (dark blue) with published datasets from hippocampus (light blue). **e**, Top 18 pathways from Reactome pathway analysis with age-related differentially methylated genes (in total 3983 genes). Pathways were ordered by significance of the

enrichment and colored based on the rank of significance. Among the top 18 enriched pathways from age-related methylation changes are also the highest enriched pathways from ENR-induced DNA methylation changes in non-aged mice after three months of ENR.

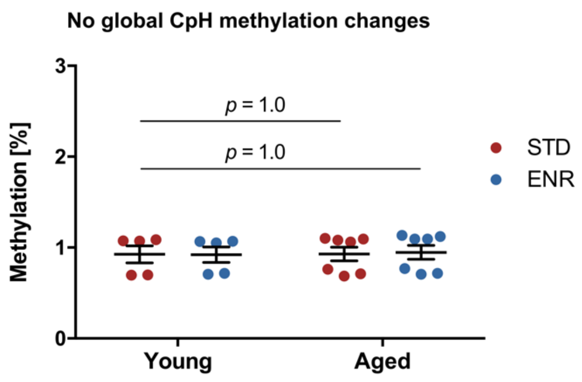

Supplementary Fig. 4

No interaction between environmental enrichment and aging in global CpH methylation. Aging and ENR do not change global CpH methylation (mean over 246,878 CpHs). The *p*-values are from two-way ANOVA with Dunnett's post hoc test.

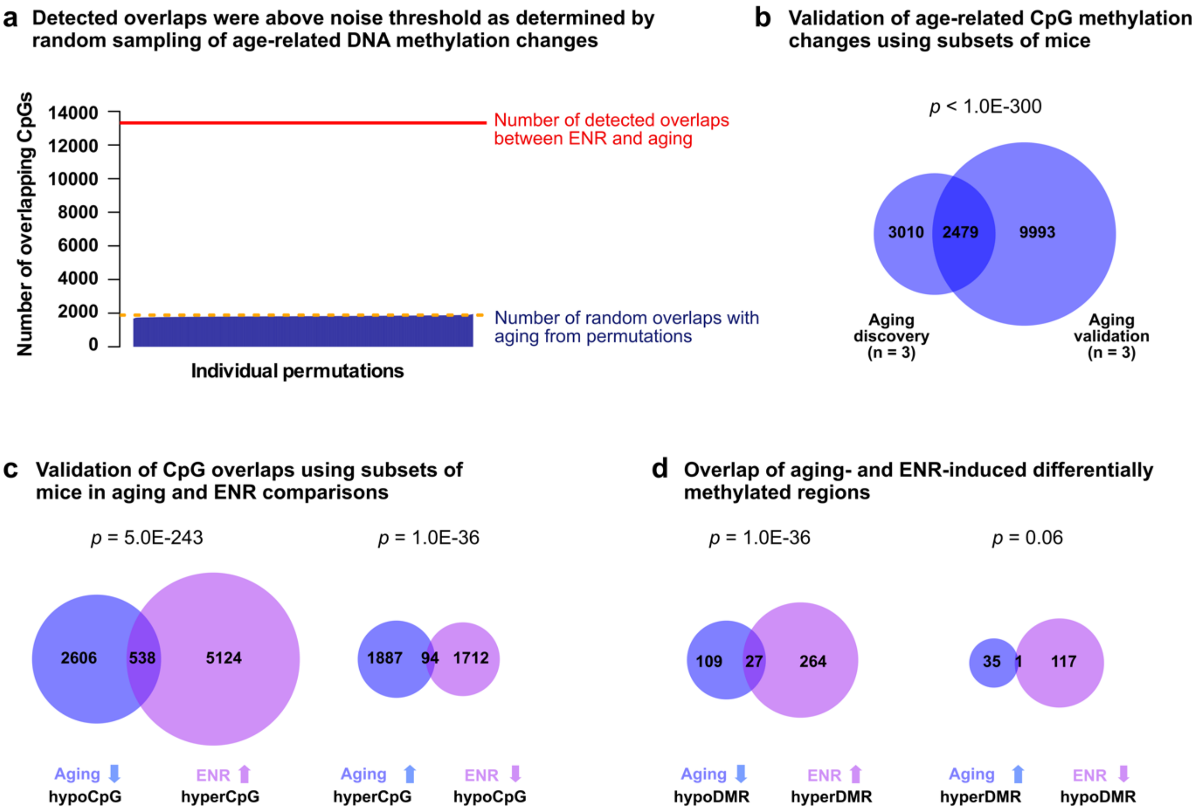

Supplementary Fig. 5

Validation of significant overlaps between age-related and ENR-induced CpG methylation changes. a, Numbers of CpGs overlapping between ENR and aging (red line) were above significance threshold (p = 0.05; dotted orange line) of background overlaps (blue). Background overlaps were determined by performing 1,000 rounds of random sampling of 33,039 CpGs (numbers of ENR-induced DNA methylation changes) from all sequenced CpGs (762,182) and calculating their overlap with age-related DNA methylation changes. **b,** Reproducibility of age-related DNA methylation changes at 45 % of individual CpGs when the group of aged STD mice was split into different discovery and validation subsets. **c,** Significant overlaps were still found when different subsets of aged STD mice were used for calculating age-related and ENR-induced CpG methylation changes. **d,** Significant overlap of age-related differentially methylated regions with ENR-induced hypermethylated regions. Hypergeometric tests were used to determine p-values in b-d.

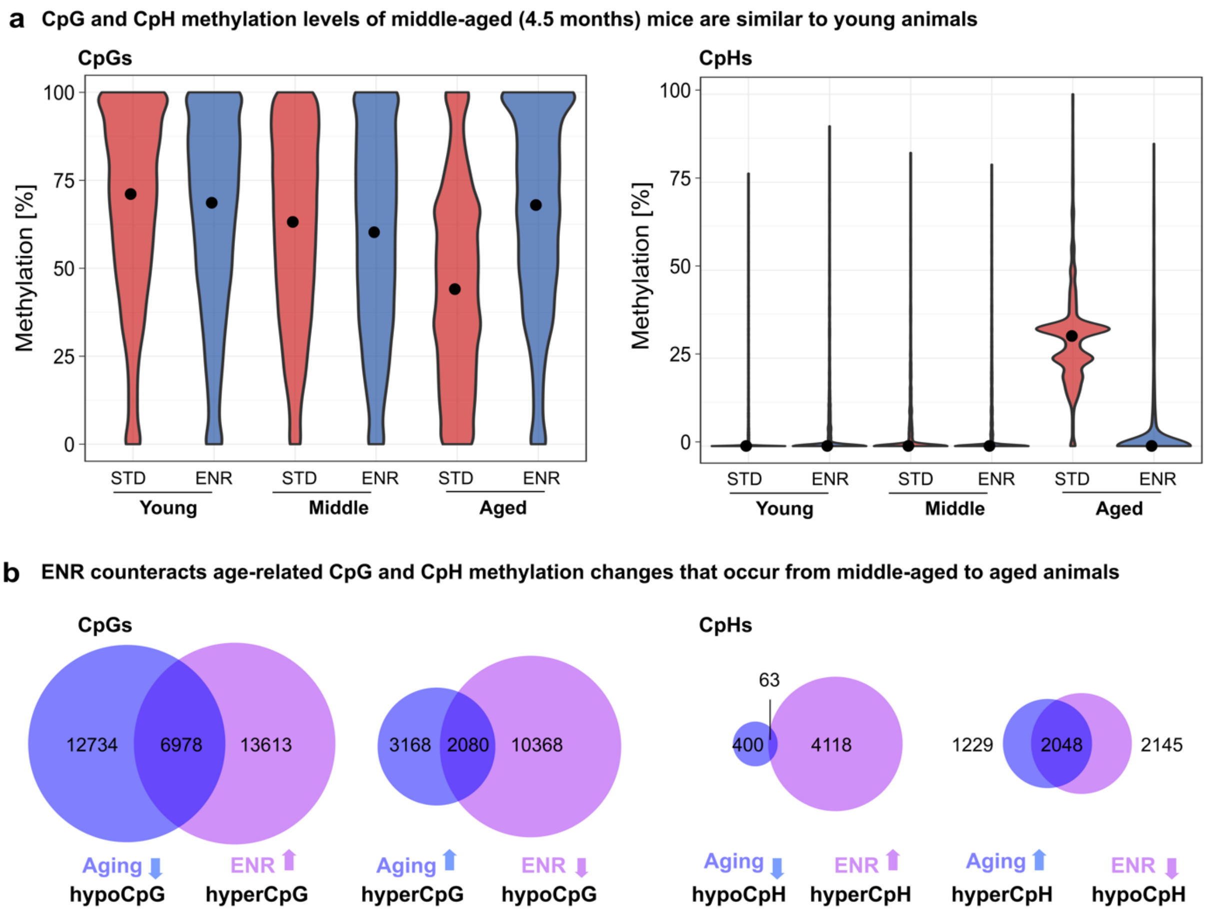

Supplementary Fig. 6

The interaction of age-related and ENR-induced DNA methylation changes between middle-aged and aged animals. a, STD and ENR housed middle-aged animals (4.5 months) show CpG and CpH methylation levels similar to young animals. While aged STD mice have significantly lower CpG methylation levels compared to young STD mice ($p = 0.0005$), no significant difference can be observed between young STD and middle-aged STD mice ($p = 0.54$; p-values from 2-way ANOVA with post hoc Tukey test; p (age) = 0.011; p (housing) = 0.086; p (interaction) = 0.0014). **b**, In total, 36.29 % of CpG methylation differences between 4.5-month-old and 14-month-old STD mice and 56.44 % of CpH methylation changes were counteracted by ENR.

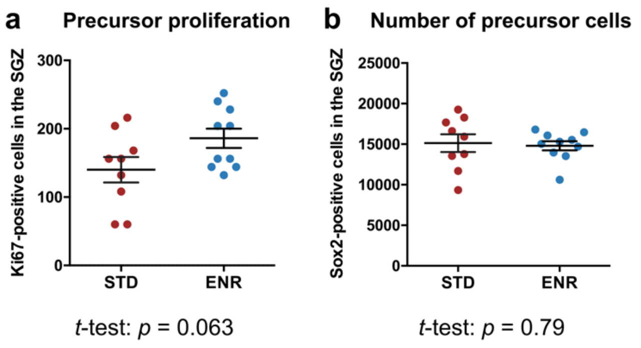

Supplementary Fig. 7

Influence of lifelong ENR on neuronal precursor cells in the dentate gyrus. **a**, Quantification of the numbers of proliferating, Ki67-positive precursor cells in the subgranular zone (SGZ). **b**, No difference in total numbers of Sox2-positive precursor cells in the SGZ between aged mice housed in STD or ENR for one year.

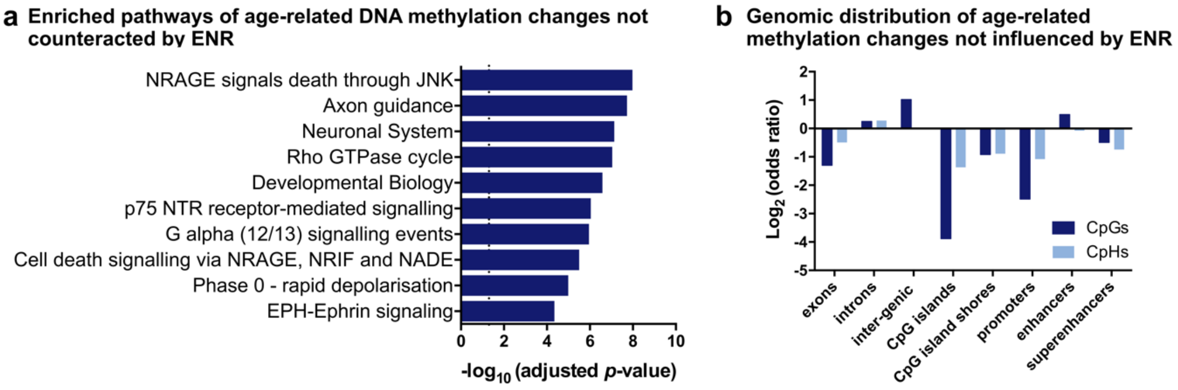

Supplementary Fig. 8

Age-related CpG and CpH methylation changes that were not counteracted by ENR showed similar functional enrichment and genomic distribution as age-related genes counteracted by ENR. a, Genes containing aging-induced CpG and CpH methylation changes that were not influenced by ENR (2,195 genes) were enriched in neuronal plasticity pathways. **b,** Age-related CpGs, but not CpHs, are significantly enriched at inter-genic regions and enhancers (adjusted $p < 0.001$).

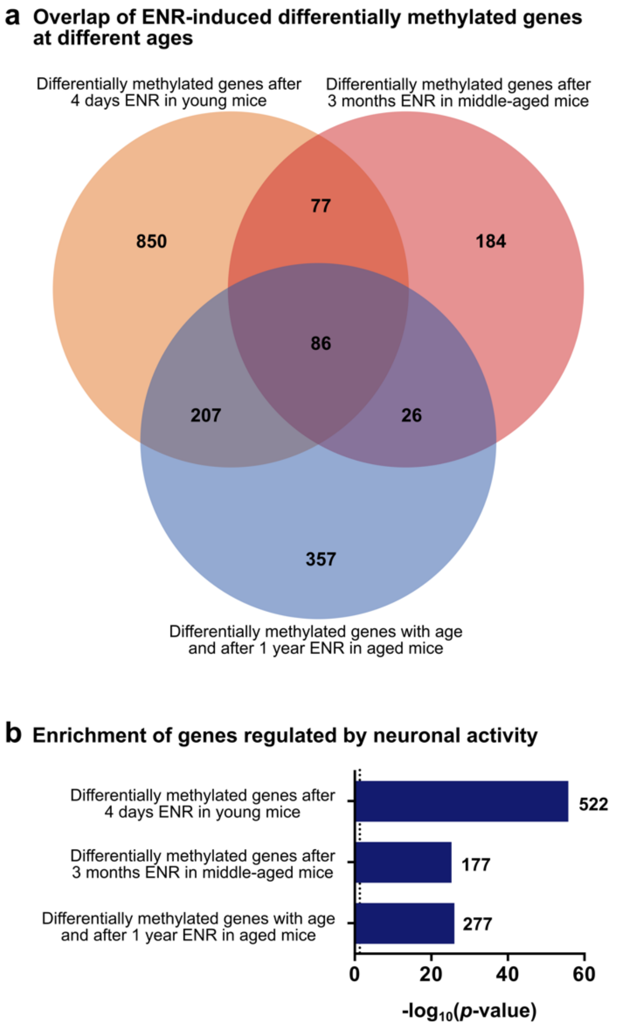

Supplementary Fig. 9

Genes at which ENR counteracted age-related DNA methylation changes overlap with ENR-induced methylation changes in the non-aged brain and with neuronal activity regulated genes. **a**, Venn diagram with number of overlapping genes between groups. **b**, Enrichment of genes that change RNA levels after activation of neurons in the dentate gyrus by electroconvulsive stimulation. Depicted are the -$\log_{10}$(p-values) and the number of overlapping genes for every dataset.

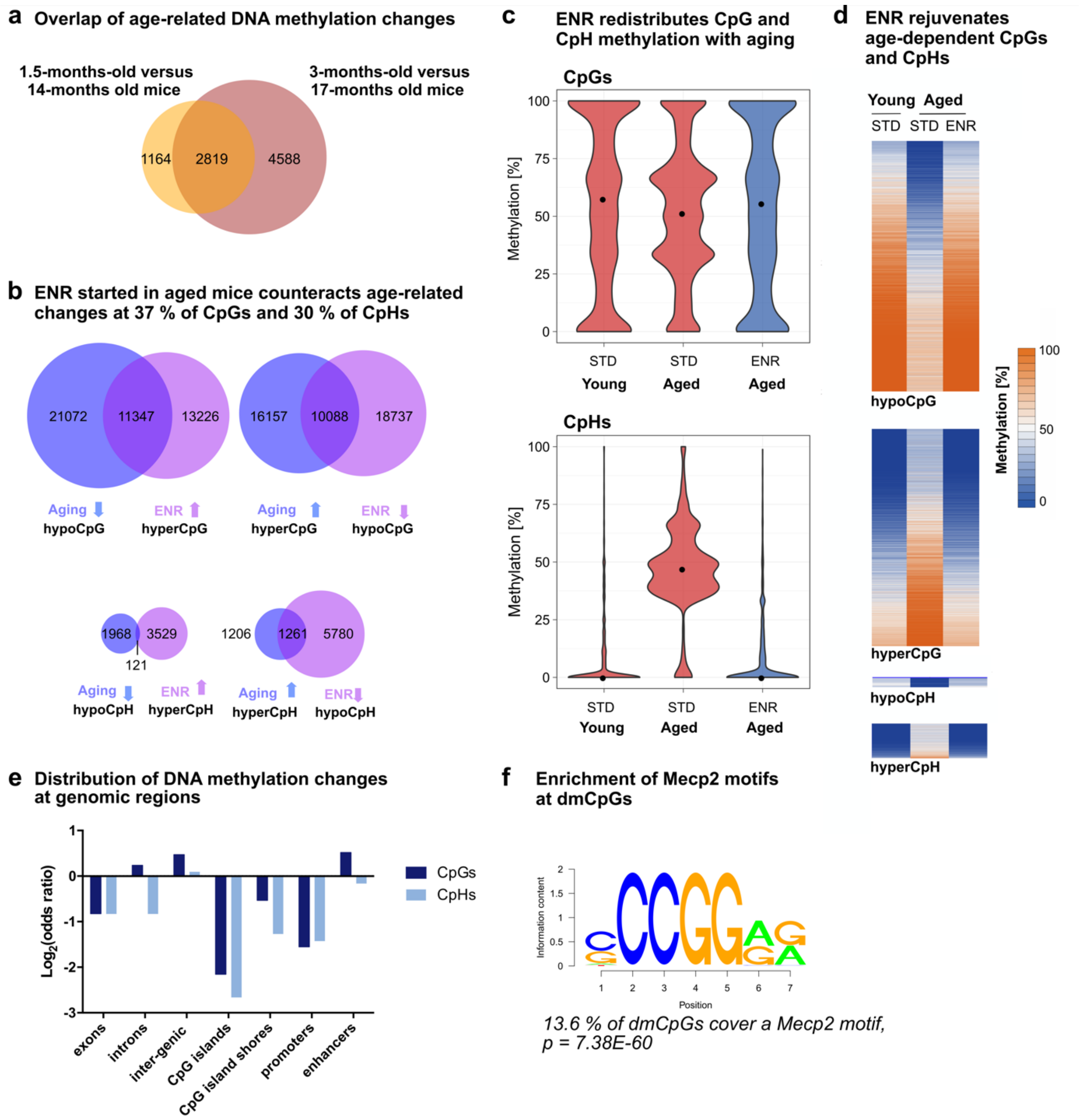

Supplementary Fig. 10

ENR housing of aged mice rescues age-related CpG and CpH methylation changes. DNA methylation profiles of 12-week-old mice (Young STD), 17-month-old mice (Aged STD) and 17-month-old mice housed in ENR for three months (Aged ENR). RRBS was performed on the dentate gyrus of eight individual mice per group. **a,** Similar age-related differentially methylated genes are detected between 12-week-old mice and 17-month-old mice (STD housed) compared with the dataset described in Supplementary Fig. 3 (1.5-month-old versus 14-month-old mice). Overlap takes direction of the DNA methylation change (hypomethylated or hypermethylated) into account. **b,** Overlap of age-related and ENR-induced DNA methylation changes (significant overlaps with $p < 1 \times 10^{-100}$ for all comparisons as determined by hypergeometric tests). **c,** Distribution of methylation percentages at the 21,435 CpGs and 1,382 CpHs at which ENR of aged mice counteracts aging effects. **d,** Absolute DNA methylation percentages (mean per group) at individual CpGs and CpHs affected differentially by age and ENR. **e,** CpGs at which three months ENR of aged mice rescued aging effects are depleted at exons, CpG islands, CpG island shores and promoters, but significantly enriched at introns, inter-genic areas and enhancers (adjusted $p < 0.001$). In contrast, CpHs were depleted at introns and not

significantly enriched at inter-genic areas and enhancers. **f,** Mecp2 motifs overlapped with 13.6 % of CpG at which ENR counteracts aging effects (hypergeometric test: adjusted $p = 7.4 \times 10^{-60}$).

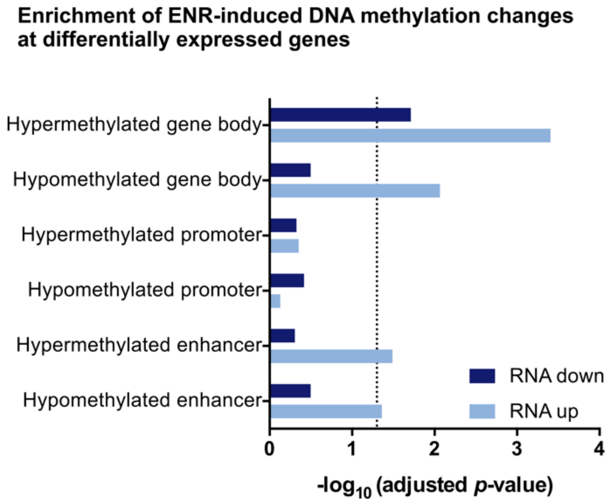

Supplementary Fig. 11

Relationship between ENR-induced DNA methylation and gene expression changes. DNA methylation changes induced by three months of ENR housing in aged mice were compared with previously published gene expression changes in response to ENR (Zhang et al., 2018). Differentially methylated genes were separated by the direction and the genomic region of the DNA methylation change and overlapped with genes up-regulated (RNA up) or down-regulated (RNA down) in response to ENR. Significant overlap was calculated using hypergeometric test with FDR correction. Dashed line indicates significance threshold of $p < 0.05$.
