# Supplementary material for: Environmental enrichment preserves a young DNA methylation landscape in the aged mouse hippocampus
Source: Nat Commun. 2021 Jun 23;12:3892. doi: 10.1038/s41467-021-23993-1 (PMC8222384; doi:10.1038/s41467-021-23993-1)
Supplement: Supplementary file 3 — Description of Additional Supplementary Files [file 41467_2021_23993_MOESM3_ESM.pdf]

Description of Additional Supplementary Files

Supplementary Data 1
Differentially methylated cytosines (sheet 1) and differentially methylated genes (sheet 2) in the dentate gyrus of 4.5-month-old mice after three months of ENR housing. Data related to Fig. 1.

Supplementary Data 2
Results of GO and pathway enrichment analyses with gene targets of ENR-induced dmCpGs in 4.5 months old mice. Listed are significantly enriched pathways of "Reactome" pathway enrichment analysis (sheet 1, Benjamini-Hochberg adjusted *q*-values), significantly enriched GO terms from GO cellular component enrichment analysis (sheet 2, FDR adjusted *q*-values), significantly enriched GO terms of SynGO biological process enrichment analysis (sheet 3, FDR adjusted *q*-values) and differentially methylated genes with functional annotation in MANGO (sheet 4) and all 373 differentially methylated genes (sheet 5). Data related to Fig. 1.

Supplementary Data 3
Lists of age-related differentially methylated cytosines in STD mice (sheet 1, SLIM adjusted *q*-values) and ENR-induced differentially methylated cytosines in aged mice (sheet 2, SLIM adjusted *q*-values). Data related to Fig. 2 and Supplementary Fig. 1.

Supplementary Data 4
Significantly enriched pathways of age-related differentially methylated genes from "Reactome" pathway enrichment analysis (Benjamini-Hochberg adjusted *q*-values). Data related to Supplementary Fig. 2.

Supplementary Data 5

Results of transcription factor motif enrichment analysis of age-related dmCpGs counteracted by ENR. Listed are significantly enriched transcription factor motifs in all 13,314 dmCpGs (sheet 1), enriched motifs in dmCpGs located within enhancers (sheet 2) and locations and methylation changes of Mecp2 target dmCpGs (sheet 3). Data related to Fig. 3.

Supplementary Data 6

Results of enrichment analysis used for generation of the enrichment map (sheet 1) and list of the 676 genes at which ENR counteracted age-related DNA methylation changes (sheet 2). Data related to Fig. 4.

Supplementary Data 7

Results of "Reactome" pathway and MANGO analyses with ENR-induced differentially methylated genes separated by genomic region and direction of the DNA methylation change (Benjamini-Hochberg adjusted *q*-values).

Supplementary Data 8

List of genes that are dysregulated with age-related cognitive decline in humans and regulated by ENR in mice (sheet 1) and results of STRING enrichment analysis (sheet 2). Data related to Fig. 5.

Supplementary Data 9

List of primer sequences used in this study.
