# Supplementary material for: Environmental enrichment preserves a young DNA methylation landscape in the aged mouse hippocampus
Source: Nat Commun. 2021 Jun 23;12:3892. doi: 10.1038/s41467-021-23993-1 (PMC8222384; doi:10.1038/s41467-021-23993-1)
Supplement: Supplementary file 13 — Reporting summary [file 41467_2021_23993_MOESM13_ESM.pdf]

nature research

Corresponding author(s): Gerd Kempermann

Last updated by author(s): Mar 23, 2021

Reporting Summary

Nature Research wishes to improve the reproducibility of the work that we publish. This form provides structure for consistency and transparency in reporting. For further information on Nature Research policies, see Authors & Referees and the Editorial Policy Checklist .

Statistics

For all statistical analyses, confirm that the following items are present in the figure legend, table legend, main text, or Methods section.

n/a	Confirmed	
☐	☒	The exact sample size (*n*) for each experimental group/condition, given as a discrete number and unit of measurement
☐	☒	A statement on whether measurements were taken from distinct samples or whether the same sample was measured repeatedly
☐	☒	The statistical test(s) used AND whether they are one- or two-sided<br>*Only common tests should be described solely by name; describe more complex techniques in the Methods section.*
☒	☐	A description of all covariates tested
☐	☒	A description of any assumptions or corrections, such as tests of normality and adjustment for multiple comparisons
☐	☒	A full description of the statistical parameters including central tendency (e.g. means) or other basic estimates (e.g. regression coefficient) AND variation (e.g. standard deviation) or associated estimates of uncertainty (e.g. confidence intervals)
☐	☒	For null hypothesis testing, the test statistic (e.g. *F*, *t*, *r*) with confidence intervals, effect sizes, degrees of freedom and *P* value noted<br>*Give P values as exact values whenever suitable.*
☒	☐	For Bayesian analysis, information on the choice of priors and Markov chain Monte Carlo settings
☒	☐	For hierarchical and complex designs, identification of the appropriate level for tests and full reporting of outcomes
☒	☐	Estimates of effect sizes (e.g. Cohen's *d*, Pearson's *r*), indicating how they were calculated

Our web collection on statistics for biologists contains articles on many of the points above.

Software and code

Policy information about availability of computer code

Data collection	No software was used for data collection.
Data analysis	Trim Galore 0.4.4, Bismark 0.19.0, methylKit 1.5.2, Genomic Ranges 3.7, AxioVision 4.8, Primer 3 0.4.0, Methprimer 1.0, DSS 2.38.0, Cytoscape 3.7.1, Prism 6.0

For manuscripts utilizing custom algorithms or software that are central to the research but not yet described in published literature, software must be made available to editors/reviewers. We strongly encourage code deposition in a community repository (e.g. GitHub). See the Nature Research guidelines for submitting code & software for further information.

Data

Policy information about availability of data

All manuscripts must include a data availability statement. This statement should provide the following information, where applicable:

- Accession codes, unique identifiers, or web links for publicly available datasets
- A list of figures that have associated raw data
- A description of any restrictions on data availability

All data have been deposited at GEO (GSE138368). Figures 1,2,4,5 contain associated raw data. There are no restrictions to data availability.

Field-specific reporting

Please select the one below that is the best fit for your research. If you are not sure, read the appropriate sections before making your selection.

☒ Life sciences ☐ Behavioural & social sciences ☐ Ecological, evolutionary & environmental sciences

Life sciences study design

All studies must disclose on these points even when the disclosure is negative.

Sample size	Sample sizes for analyzing hippocampal neurogenesis by immunohistochemistry were calculated using effect sizes from a previous publication (PMID 30362941). Sample sizes for RRBS were chosen based on previously published experiments that showed DNA methylation changes after behavioral interventions (PMIDs 29352183, 26656643). No calculation to determine sample size was performed for sequencing experiments.
Data exclusions	No data were excluded from analyses.
Replication	DNA methylation changes at individual genes were replicated by targeted bisulfite sequencing and RRBS. Sample sizes for RRBS and targeted bisulfite sequencing were chosen based on previously published experiments that showed DNA methylation changes after behavioral interventions (PMIDs 29352183, 26656643).
Randomization	Mice were randomly assigned to live in environmental enrichment or standard housing.
Blinding	The experimenter was blinded during DNA isolation, preparation of RRBS libraries and sequencing, immunoprecipitation, and during immunohistochemistry and quantification of hippocampal neurogenesis. No blinding was performed during RNA isolation, cDNA synthesis and preparation of qPCR reactions.

Reporting for specific materials, systems and methods

We require information from authors about some types of materials, experimental systems and methods used in many studies. Here, indicate whether each material, system or method listed is relevant to your study. If you are not sure if a list item applies to your research, read the appropriate section before selecting a response.

Materials & experimental systems

n/a	Involved in the study	
☐	☒	Antibodies
☒	☐	Eukaryotic cell lines
☒	☐	Palaeontology
☐	☒	Animals and other organisms
☒	☐	Human research participants
☒	☐	Clinical data

Methods

n/a	Involved in the study	
☒	☐	ChIP-seq
☒	☐	Flow cytometry
☒	☐	MRI-based neuroimaging

Antibodies

Antibodies used	Anti-doublecortin (Santa Cruz, sc-8067); Anti-Sox2 (Santa Cruz, sc-17320), Anti-Ki67 (eBioscience, 14569882), Anti-Mecp2 (Diagenode, C15410052), Alexa Fluor 488 Donkey Anti-Rat IgG (Jackson ImmunoResearch, 712-545-153), Cy3 Donkey Anti-Goat IgG (Jackson ImmunoResearch, 705-165-151).
Validation	All antibodies were used and validated in previous studies (see PMIDs 27050949, 30905740, 25555543, 27008915).

Animals and other organisms

Policy information about studies involving animals; ARRIVE guidelines recommended for reporting animal research

Laboratory animals	Female C57Bl6JRj, age six weeks to 17 months. Mice were maintained on a 12 h light/dark cycle, at a temperature of 23°C +/- 1°C with 40-60 % humidity. Food and water were provided ad libitum. Mouse in control cages lived in groups of 5 animals per cage (Type II, Tecniplast). Mice in enriched environments (a 0.74 m2 enclosure equipped with tunnels and plastic toys) were housed in groups of 10 mice.
Wild animals	The study did not involve wild animals.
Field-collected samples	The study did not involve samples collected from the field.
Ethics oversight	Ethics oversight was provided by the animal welfare officer of the Technische Universität Dresden and the Landesdirektion Sachsen.

Note that full information on the approval of the study protocol must also be provided in the manuscript.
